# Unusual metastases of melanoma

**DOI:** 10.1002/ccr3.1732

**Published:** 2018-07-23

**Authors:** Guido Carillio, Stefano Molica

**Affiliations:** ^1^ Department of Oncology and Hematology “Pugliese‐Ciaccio” Hospital Catanzaro Italy

**Keywords:** cardiac metastasis, immune checkpoint inhibitor, malignant melanoma

## Abstract

Malignant melanoma is a very aggressive tumor. Immune and targeted therapy could prolong patient's clinical benefit and survival, but the correct sequence of therapies has still to be defined.

## 1 INTRODUCTION

Metastatic melanoma remains a challenging condition, causing rapid death. Novel treatment strategies, such as immune and targeted therapy, could prolong patient's clinical benefit and survival, even in cases with apparent poor prognosis. The correct sequence of therapies has still to be defined.

## 2 QUESTION

Which sequence of therapies does ensure the best quality of life and survival in patients affected by BRAF‐mutated metastatic melanoma?

## 3 DISCUSSION

**Figure 1:**
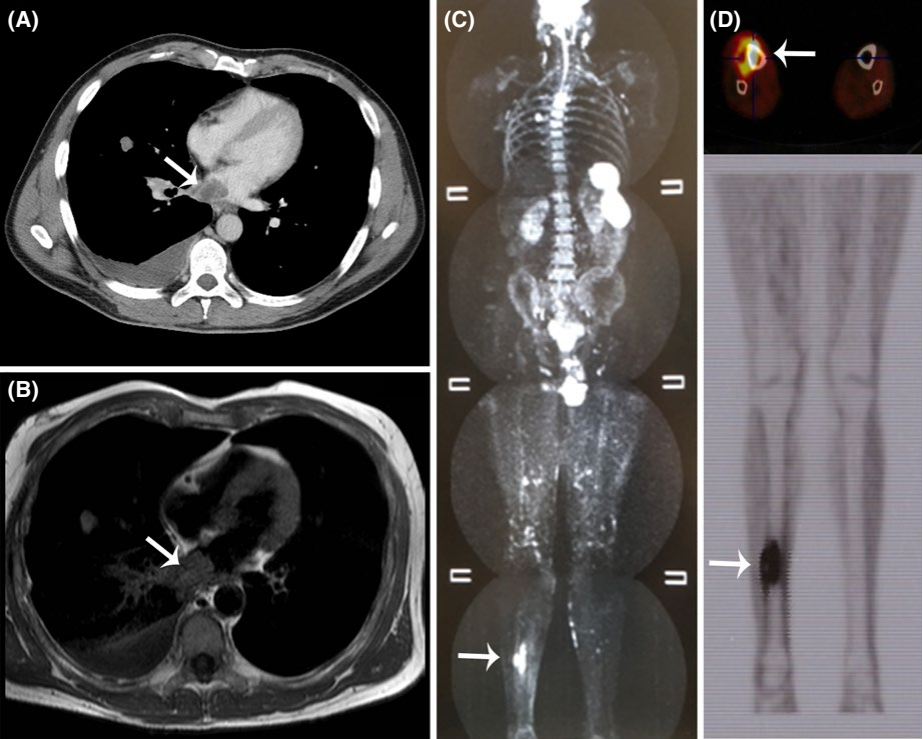
CT (Panel A) and MRI scan (Panel B) showing a metastasis in the left cardiac atrium; whole body MRI (Panel C) and axial/coronal PET scan (Panel D) detecting a metastasis in the right tibia

A 50‐year‐old man was admitted to our hospital for asymptomatic brain, lung, and subcutaneous metastases of malignant melanoma. Biomolecular characterization of primary tumor was not available. Up‐front ipilimumab followed by whole brain radiotherapy was given, with the aim of favoring radiotherapy abscopal effect.^1^ Post‐treatment CT scan (Figure 1, Panel A) revealed stable disease at known sites, but appearance of metastasis in the left cardiac atrium, confirmed by MRI scan (Panel B and Video S1). Cardiosurgical excision of the lesion was successfully performed and permitted to detect BRAF V600E mutation. Thus, single agent vemurafenib was effectively administered for 13 months, until an unusual right tibial metastasis was documented by whole body MRI and PET scan (Panels C and D). Hypofractionated bone radiotherapy and third‐line pembrolizumab were administered. An initial pseudoprogression was diagnosed by CT scan, with the patient maintaining clinical benefit. Therefore, the immune checkpoint inhibitor was continued up to 14 months, obtaining durable stable disease and good quality of life. A final severe encephalic progression, uselessly treated with temozolomide and re‐challenging ipilimumab,^2^ caused patient's death after 3 years from the first‐line therapy. In our experience, selected patients with BRAF‐mutated melanoma and without imminent risk of death can be suitably treated with up‐front immunotherapy followed by targeted therapy.

## CONFLICT OF INTEREST

None declared.

## AUTHORSHIP

GC: wrote the paper and participated in the management of patient care. SM: guided treatment decisions and participated in the management of patient care.
